# Bilateral Ovarian Cystadenoma in a Dog: Morphological and Histopathological Features

**DOI:** 10.1155/crve/8710723

**Published:** 2026-08-19

**Authors:** Zahra Nozari, Samin Khodaverdian, Asghar Mogheiseh, Nasrollah Ahmadi, Shiva Amanollahi

**Affiliations:** ^1^ Department of Clinical Sciences, School of Veterinary Medicine, Shiraz University, Shiraz, Fars, Iran, shirazu.ac.ir; ^2^ Department of Pathobiology, School of Veterinary Medicine, Shiraz University, Shiraz, Fars, Iran, shirazu.ac.ir

**Keywords:** estrus, fertility, histopathology, multiple cysts, ovaries, ultrasound

## Abstract

Normal and abnormal cystic structures around the ovaries need to be evaluated based on the history, clinical signs, diagnostic imaging and paraclinical methods. An intact spitz nulliparous female, 9 years old, was referred with a normal estrous cycle and health history. During the ultrasound examination, bilateral cystic structures were observed around the ovaries just caudal to the kidneys. The left‐side cystic masses were larger than the right‐side and no abdominal free fluid observed. Based on the cystic structures and the age of the dog, an ovariohysterectomy was performed and histopathological samples were prepared for evaluation. Histopathological evaluation revealed multicystadenoma tumors originating from the subepithelial structures of the ovaries. No clinical signs of hormonal activity of cystic tumors were observed. They may affect transportation of oocytes from the ovary to the oviduct due to their physical structures in the ovulation space and infundibulum of the oviduct.

## 1. Introduction

Cystic structures are fluid‐filled compartments that develop within or on the surface of ovaries in dogs and cats. These cystic formations constitute a significant clinical entity in small animal reproduction, which untreated often lead to infertility [1]. Cysts from various structures occur in and external to the canine ovary. In animals, cysts occurring within the ovary arise from subsurface epithelial structures (SES), graafian follicle cysts and cystic rete ovarii. Cysts falling out the ovary include the cysts coming from remnants of wolffian and/or Mullerian tubules or ducts [2]. However, histology is used to distinguish different types of cystic structures of ovary [3].

The incidence of ovarian tumors in intact female dogs is relatively low, estimated at 6.25%, and constitutes approximately 0.5%–1.2% of all canine neoplasia. These tumors are typically found in older female dogs or in those with ovarian remnant syndrome. Ovarian neoplasms have been diagnosed in dogs ranging from 14 months to 16 years of age, with the majority of cases observed in dogs older than 10 years. Notably, granulosa cell tumors (GCTs) and teratomas tend to manifest at a slightly younger age specific breeds, including German shepherds, boxers, Yorkshire terriers, poodles, Boston terriers, and English bulldogs, exhibit a higher incidence [4]. Canine ovarian neoplasms are classified according to their histogenetic criteria into epithelial, germ cell, sex cord stromal, and mesenchymal tumors. Epithelial tumors are diagnosed in almost 50% of the dogs with an ovarian tumor. Papillary adenocarcinomas or cystic adenocarcinomas represent more than 60% of them [5].

Literature related to ovarian cysts and neoplasia in dogs was assessed to summarize studies and case series on ovarian cysts and primary ovarian tumors in regard to epidemiologic, clinical and fertility aspects. Ovarian disease in female dogs is uncommon but may affect health, fertility, or even the life of the animal [6]. We are aimed at reporting the presence of bilateral multiple cystic structures diagnosed as cystadenoma in the ovaries of a mixed breed dog. These structures were incidentally observed during ovariohysterectomy.

## 2. Case History

A spitz nulliparous female, 9 years old, was referred to the educational veterinary hospital at the School of Veterinary Medicine, Shiraz University. The owner reported a regular estrous cycle every 6 months, with the last proestrus occurring about 2 months ago. The main complaint of the owner was a small swelling in front of the umbilicus that appeared after surgery to repair an umbilical hernia. As part of a routine examination for unspayed geriatrics, an abdominal ultrasound was performed. The uterus appeared normal, but despite the history of a regular estrous cycle, bilateral multicystic structures were observed in the ovarian location just caudal to the kidneys.

## 3. Clinical Findings

**Figure 1:**
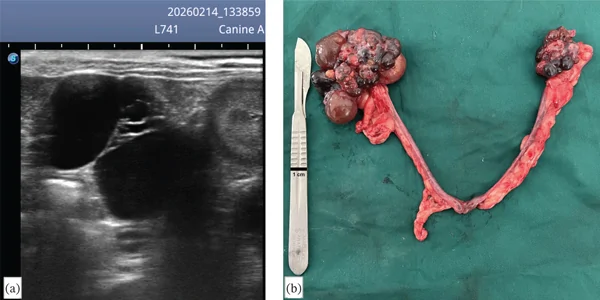
(a) Ultrasound view of cystic structures observed just caudal to the kidney. (b) Gross morphology of left‐ and right‐side multiple cystic structures around the ovaries of an intact spitz nulliparous female, 9 years old. Left‐side structures are greater than the right side and uterus is in normal condition.

Ultrasound examination revealed multicystic structures located in the ovary area, caudal to the kidneys. The cystic structures were 1.71 ± 0.63 (0.67–2.7 cm) in diameter (Figure 1a). The uterus showed normal echotexture and echogenicity, consistent with the anestrus phase of the estrous cycle. No abdominal free fluid was observed, and the kidneys and other nearby structures appeared normal in size, echogenicity, and echotexture. Vaginal cytology indicated the anestrus phase of the estrous cycle, with no abnormal secretions observed.

Following ovariohysterectomy and gross examination, multiple cystic structures were found covering both ovaries, whereas the uterus appeared normal. The cystic mass on the left side was 6.23 × 4.26 cm, and the one on the right was 2.91 × 2.75 cm (Figure 1b). The ovaries and uterus were sent to the histopathology laboratory for further evaluation.

## 4. Histopathological Evaluations

For histopathological examination, representative samples of both ovaries and uterine tissues were obtained and immediately placed in 10% neutral buffered formalin (NBF). After 24 h, the fixative solution was changed. Once complete fixation was achieved, the tissue specimen was embedded in paraffin. Tissue processing steps, including dehydration, clearing, and impregnation, were performed using the Autotechnicon tissue processor. Subsequently, tissue sections with a thickness of 5 *μ*m were prepared using a microtome. Finally, the slide containing the tissue section was stained with hematoxylin and eosin (H&E) and examined under a light microscope (Olympus, CX21FS1, Japan) [7].

## 5. Histopathological Findings

**Figure 2:**
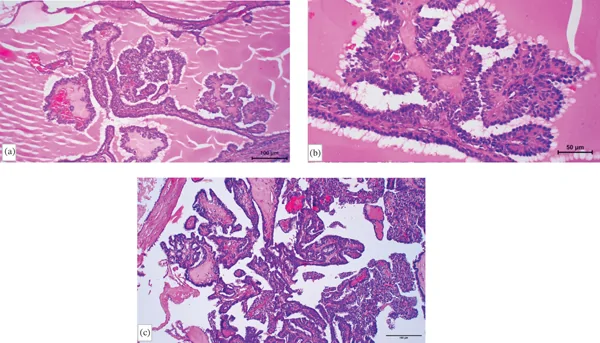
(a) Serous ovarian cystadenoma as an epithelial tumor in a dog. The photomicrograph showing the benign proliferations of epithelial cells with arboriform papillary pattern projecting into the lumen of serous cystic cavities with eosinophilic proteinaceous materials (H&E stain, scale bar = 100 *μ*m). (b). Higher magnification of the serous cystadenoma of the ovaries showing focal proliferation of nonciliated pseudostratified cuboidal or columnar epithelial cells containing oval or round nuclei and distinct and homogenous cytoplasm (H&E stain, scale bar = 50 *μ*m). (c) The photomicrograph of a serous ovarian cystadenoma as an epithelial tumor in the right ovary of a dog showing the papillary proliferations of nonciliated pseudostratified cuboidal or columnar epithelial cells with containing oval or round nuclei and distinct and homogenous cytoplasm (H&E stain, scale bar = 100 *μ*m).

Histopathological examination of the H&E‐stained tissue sections of both ovaries revealed the benign proliferations of epithelial cells in the ovarian tissue with arboriform papillary pattern projecting into the lumen of many serous cystic cavities containing eosinophilic proteinaceous materials (Figure 2a,c). The neoplastic nonciliated pseudostratified cuboidal or columnar epithelial cells had oval or round nuclei and distinct homogenous cytoplasm (Figure 2b,c). Also, there was no tumor cell invasion to the ovarian capsule, stroma, and blood vessels. Based on the above microscopic descriptions, the neoplastic mass was suggestive of a serous ovarian cystadenoma.

## 6. Discussion

Ovarian diseases in bitches present a diagnostic and clinical challenge due to their diverse etiologies and variable clinical manifestations [1]. Clinical signs are primarily determined by the hormonal activity of the lesion and its physical mass effect, often manifesting as fertility issues, including abnormal estrous cycles, breeding failures, or pregnancy complications [8]. Although cystic structures in and around the ovaries are common, particularly in older animals, their diagnosis and management remain areas of significant clinical uncertainty. A notable gap exists in standardized diagnostic protocols; current literature focuses predominantly on surgical intervention (ovariohysterectomy), with limited exploration of nonsurgical treatment options and underutilization of crucial diagnostic tools such as hormone profiling [9]. Advancing our understanding of cyst pathogenesis, early detection, and fertility‐sparing management is essential for improving animal welfare and enabling informed clinical decision‐making, particularly in breeding animals [1, 8].

Functional ovarian cysts are fluid‐filled structures that may be solitary or multiple, unilateral or bilateral, and typically affect bitches between 6 and 8 years of age [8]. Although their exact pathogenesis remains obscure, it is hypothesized that an insufficient luteinizing hormone (LH) surge, along with intrafollicular alterations in gonadotrophin receptors and growth factors, contributes to the development of hormonally active cysts [4]. These cysts are clinically significant as they predispose bitches to the cystic endometrial hyperplasia–pyometra complex and, in some cases, hyperestrogenism [8, 10]. Given the high likelihood of concurrent uterine pathology at diagnosis, ovariohysterectomy remains the standard treatment for follicular and luteal cysts [8].

The multifactorial nature of follicular growth and maturation, involving complex cellular differentiation processes, likely explains the varying prevalence of different cyst types—a finding consistent with clinical observations in bitches presenting with gynecopathies [3]. Furthermore, a documented high prevalence of hypothyroidism in bitches with ovarian cysts suggests a potential endocrine axis disruption. Given the established impact of thyroid function on ovulation in other species, thyroid function testing is recommended as part of the diagnostic workup for infertile bitches [11, 12]. Parallels can also be drawn to human medicine, where polycystic ovary syndrome (PCOS) is a leading cause of anovulatory infertility, often associated with hyperinsulinemia and insulin resistance—conditions with both reproductive and long‐term cardiovascular implications [11].

Ultrasonography serves as the primary imaging modality for evaluating ovarian structures [13]. Normal ovarian morphology varies cyclically; during anestrus, for example, ovaries appear as small, homogeneous, and oval structures. Pathologic ovarian cysts are defined as persistent cystic structures within the ovarian parenchyma that correlate with clinical signs of abnormal hormonal effects (estrogen or progesterone excess) [8]. These cysts represent approximately 80% of ovarian diseases and encompass a range of entities, including cystic rete ovarii, follicular, and luteinized cysts [4]. In ultrasound imaging, normal physiological follicles typically do not exceed 5–8 mm in diameter [8, 13]. Endocrine‐active cysts are of particular clinical relevance, often resulting in persistent estrus, irregular cyclicity, and uterine pathologies such as cystic endometrial hyperplasia and pyometra [10]. When ultrasonographic features deviate from normal cyclic variations or typical benign cystic disease, early‐stage ovarian tumors must be considered as a primary differential. However, this distinction is critical, as it influences treatment planning and prognosis [6].

Ovarian tumors in dogs are relatively uncommon, largely due to the widespread practice of elective ovariohysterectomy. When they do occur, they predominantly affect older, intact females, with an overall prevalence of 0.5%–1.2%, rising to 6.25% in unspayed populations [14]. A comprehensive evaluation of gross pathology in conjunction with endocrinology is essential, as previous reports have often been limited to small case series, potentially biasing perceptions of disease presentation [3]. Epithelial tumors are the most common ovarian neoplasms, accounting for 65% of cases, with cystadenomas being the most frequent subtype. These tumors typically present as unilateral, multicystic structures filled with serous fluid, often resembling a cluster of thin‐walled, grape‐like cysts. Although benign cystadenomas are prevalent, malignant cystadenocarcinomas, though rare, are clinically significant due to their metastatic potential and require complete surgical excision [14].

The sex cord stromal tumors include GCTs, luteomas, and thecomas. GCTs, the most common sex cord‐stromal tumor, may appear as solid, lobular masses or as solitary large cysts often containing bloody fluid [14]. Luteomas are rare, hormonally active tumors that can secrete estrogen or progesterone, leading to endometrial hyperplasia or other hormonal sequelae [10]. The histological diversity within this group complicates differentiation, necessitating immunohistochemical support in some cases. For instance, cysts of SES have been shown to exhibit a positive immunoreaction for placental alkaline phosphatase (PLAP), suggesting its utility as a diagnostic marker [2].

A definitive diagnosis should integrate multiple data streams, including a thorough reproductive history, physical examination, advanced imaging, and, where indicated, hormonal assays. The anatomical location of the ovaries, enclosed within the ovarian bursa and adjacent to the ipsilateral kidney, can complicate imaging. Large ovarian masses may occupy a significant portion of the abdominal cavity, making identification of the organ of origin challenging [15]. In such cases, computed tomography (CT) is invaluable. A consistent CT finding in ovarian tumors is a convoluted, tortuous ovarian artery, which serves as a reliable landmark for identifying the ovarian origin of a large abdominal mass—analogous to the use of the tortuous pampiniform plexus in identifying retained testicles [15].

Nonneoplastic cystic structures, such as ovarian rete cysts (clusters of small cysts within the ovarian hilus) and paraovarian cysts (thin‐walled cysts in the mesovarium), are often incidental findings [2, 3]. Differentiating these from neoplastic epithelial cysts is crucial, as the latter have greater clinical implications. The current body of knowledge on canine ovarian cystic structures is hampered by a lack of standardized diagnostic criteria and a reliance on surgical treatment paradigms. Key research priorities include: Elucidating the cellular and molecular changes within the hypothalamo‐hypophyseal‐ovarian axis that drive cyst formation. Developing and validating nonsurgical treatment options, particularly for breeding animals where fertility preservation is paramount [1, 9]. Establishing standardized diagnostic protocols that integrate ultrasonography, hormonal profiling (including thyroid and sex hormones), and, when necessary, advanced imaging.

Our findings contribute to this evolving field by highlighting that cystic structures can vary widely in size—an important consideration for clinicians using imaging [13]. Furthermore, the observation that fertile dogs have a significantly higher prevalence of cysts and tumors supports the hypothesis that prolonged, uninterrupted estrous cycles may increase ovarian exposure to hormonal stimuli that drive pathology [4].

## 7. Conclusion

Canine ovarian cystic structures encompass a broad spectrum of conditions, from benign functional cysts to malignant neoplasms. Accurate diagnosis requires a systematic approach integrating clinical history, imaging, and endocrinology. Although ovariohysterectomy remains the definitive treatment for many conditions, particularly when uterine pathology is present, there is a pressing need for research into nonsurgical management strategies. A deeper understanding of the pathogenesis of these conditions, coupled with improved diagnostic precision, will enable more informed clinical decisions, ultimately enhancing the health and fertility of bitches. In this report, the thyroid and reproductive hormones were not measured and limited the conclusions on the function of cystic structures of ovaries. The immunohistochemistry staining such as PALP was not performed that could confirmed tumors originated from SES.

## Funding

This study was supported by the School of Veterinary Science, Shiraz University (10.13039/100009587) (5RLG1M154630).

## Ethics Statement

All applicable international, national, and/or institutional guidelines for the care and use of animals were followed.

## Conflicts of Interest

The authors declare no conflicts of interest.

## Data Availability

The data that support the findings of this study are available from the corresponding author upon reasonable request.
